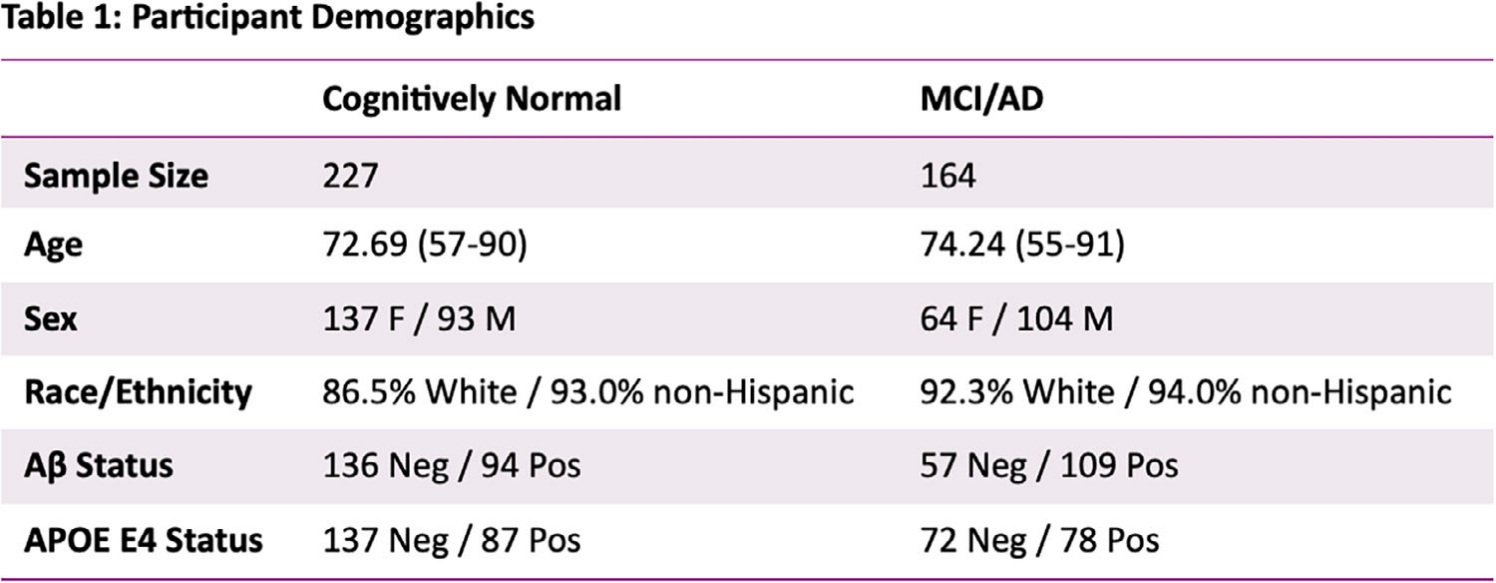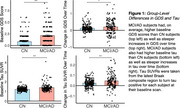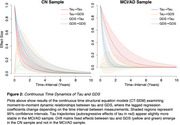# Continuous Time Dynamics of Tau and Depressive Symptoms Across the Alzheimer’s Disease Continuum

**DOI:** 10.1002/alz70861_108212

**Published:** 2025-12-23

**Authors:** Teodora Z. Markova, Jacob M. Hooker, Anne S. Berry

**Affiliations:** ^1^ Brandeis University, Waltham, MA USA; ^2^ Martinos Center for Biomedical Imaging/Harvard Medical School/Massachusetts General Hospital, Charlestown, MA USA

## Abstract

**Background:**

Recent evidence suggests that early accumulation of tau in brainstem neuromodulator‐producing nuclei may contribute to neuropsychiatric symptoms (NPS) in aging and preclinical Alzheimer’s Disease (AD). We examined temporal dynamics between tau and NPS to better understand their progression across the AD continuum.

**Methods:**

We used data from the Alzheimer’s Disease Neuroimaging Initiative, including 227 cognitively normal (CN) and 164 MCI/AD subjects with at least two timepoints of tau PET ([18‐F]Flortaucipir) and Geriatric Depression Scale (GDS) data. Subject‐specific tau ROIs were defined by the latest Braak composite region to become tau positive. Longitudinal dynamics between tau and GDS were modeled using the *ctsem* package in R.

**Results:**

The MCI/AD group had higher baseline GDS (t(385)=7.83, *p* <.001) and tau (t(385)=5.41, *p* <.001), and steeper longitudinal increases in both GDS (t(385)=2.02, *p* =.045) and tau (t(385)=4.01, *p* <.001) over time. We did not observe direct relationships between cross‐sectional or longitudinal tau and GDS in either sample (all *p* >.203). In CN subjects, elevated tau predicted higher GDS at later timepoints (drift matrix fixed effects=1.323, 95% CI (0.658, 2.032)) and higher GDS predicted higher tau at later timepoints, though to a lesser extent (0.429, 95% CI (0.222, 0.623)). Both effects extended up to ∼4 years. No such relationships emerged in the MCI/AD sample.

**Conclusions:**

In CN older adults, tau accumulation drives worsening depressive symptoms, while higher depressive symptoms in turn facilitate tau accumulation, creating a maladaptive loop. These findings support models proposing that early tau‐related disruptions in cognitively unimpaired older adults may contribute to emerging NPS.